# Integrative Medicine in the Canadian Medical Profession: Certificate of Added Competence Proposal for Physicians

**DOI:** 10.1177/27536130231182426

**Published:** 2023-06-26

**Authors:** Esther Konigsberg

**Affiliations:** 1 Department of Family Medicine, 3710 McMaster University, Hamilton, ON, Canada

**Keywords:** integrative medicine, education, functional medicine, health policy

## Abstract

Most Canadians use some form of Complementary and Alternative Medicine (CAM) and most Canadian physicians are not able to address their patients’ use due to inadequate training. Integrative Medicine (IM) in the medical profession has grown over the last 20 years and is now recognized as a sub-specialty in the United States. Canada is lagging behind. The current state of CAM and IM education for physicians in Canada is described, using the United States’ experience in comparison. The landscape and obstacles for Integrative Medicine for Canadian physicians is reviewed. A case is made for recognition of Integrative Medicine by Canadian Medical Colleges in order to advance this field in Canada.

In a 1996 British Medicine Journal (BMJ) editorial, David Sackett, a Canadian medical doctor and pioneer in evidence-based medicine wrote that “the practice of evidence-based medicine means integrating individual clinical expertise [with] the best available external clinical evidence from systematic research… with patient preference.” Integrative medicine (IM) is the medical term used when complementary and alternative medicine (CAM) therapies are integrated with conventional medical care. Many Canadians now use CAM therapies,^1^ even though their medical doctors often lack exposure to these therapies,^2,3^ due in part to limited medical education opportunities while in medical school and subsequently in post-graduate medical education. Canadian medical doctors need to be aware of IM evidence to effectively manage the care of their patients.^4^ In contrast, many European countries and the United States (U.S.) have established educational and certification programs for medical doctors in IM to inform them about IM evidence and guide the integration of CAM therapies into the comprehensive delivery of healthcare.

This article will describe the current state of CAM and IM in the medical profession in Canada and use the United States’ experience in comparison. A case will be made for recognition of Integrative Medicine by Canadian Medical Colleges.

## Background

The interest in IM has grown in the medical profession around the world. The U.S. has been a world leader in developing education in Integrative Medicine.^5^

• Integ**rative Medicine Fellowships and Residencies.** The University of Arizona’s (U of A) Center for Integrative Medicine fellowship, established in 1997, was the first IM Fellowship in the US. Currently there are eighteen IM fellowships based in the U.S. Most of the fellowships are on-site or hybrid, some offering a predominantly virtual curriculum. Approximately 2200 MDs have graduated from these fellowships to date. The U of A Center for Integrative Medicine also created the first IM curriculum for residency education, “Integrative Medicine in Residency” (IMR) which began in 2008.^6^ This curriculum has been added to 85 residency programs in the United States including Family Medicine, Internal Medicine, Pediatrics, Psychiatry, and Obstetrics and Gynecology. To date there have been a total of 1130 Resident Graduates as well as 1142 Current Residents.
• **Integrative Medicine Associations: Academic Consortium for Integrative Medicine and Health** (ACIMH) which began in 1999. The ACIMH is a consortium of more than 75 academic medical centres, nursing schools and health systems from the United States, Canada, Australia, Brazil and Mexico. The five Canadian member institutions are: University of Alberta, University of Calgary, McMaster University, University of Toronto, and University of Saskatchewan. ACIMH members commonly participate in multi-centered IM research projects which often lead to evidence-based clinical guideline development. Members also participate in international conferences and share educational initiatives. The ACIMH has stated that “integrative medicine and health reaffirms the importance of the relationship between practitioner and patient, focuses on the whole person, is informed by evidence, and makes use of all appropriate therapeutic and lifestyle approaches, healthcare professionals and disciplines to achieve optimal health and healing.”
• **Integrative Medicine Certification**: Medical fellows who have graduated, are eligible to take examinations for board certification in Integrative Medicine. The American Board of Integrative Medicine (ABOIM) was established in 2013 as a specialty under the American Board of Physician Specialties. Many IM physicians believe that board certification adds credibility and distinguishes them as having added competency in the field of Integrative Medicine when compared with other providers. There has been a total of 990 ABOIM diplomates.
• Integrative Medicine at the **National Institutes of Health** (NIH) in the U.S. The NIH opened the “Office of Alternative Medicine” in 1992. In 2014 it was renamed The National Center for Complementary and Integrative Health*”* (NCCIH) and is focused on research in integrative medicine. The NCCIH states that: “Integrative health aims for well-coordinated care among different providers and institutions by bringing conventional and complementary approaches together to care for the whole person.”^7^
• F**unctional Medicine.** Additional IM training is also available through the Institute for Functional Medicine (IFM) which states that functional medicine is an individualized, patient-centered, and science-based approach that promotes optimal wellness and addresses the underlying causes of disease.^8^ 490 US MDs have completed functional medicine training and have been certified by IFM. Some medical doctors have completed both IFM and IM fellowship trainings.

**Canadian patients** were surveyed in 2016 by the Fraser Institute (Complementary and Alternative Medicine: Use and Public Attitudes 1997, 2006, and 2016^1^). The survey noted that seventy-nine percent of Canadians had used a CAM therapy and more than half (56%) of Canadians had used a CAM therapy in the year prior to the survey. Even though the majority of people choosing CAM therapies did so for “wellness” over one-half of the visits also addressed medical concerns. Surveyed Canadians were interested in IM, the integration of conventional medicine and CAM.

### IM in the Canadian Medical Profession

• **Canadian Physicians**. As of June 2021, 101 Family Practice residents had completed the IMR and 35 residents were currently enrolled. 52 Canadian Physicians have completed an IM fellowship in the United States, 6 are currently enrolled. There are currently seven Canadian physicians certified by ABOIM in the United States. To qualify they must have graduated from a medical school and residency program through either the Royal College of Physicians and Surgeons of Canada or the College of Family Physicians of Canada, are licensed to practice medicine, and have completed an ABOIM approved Fellowship in IM. Some Canadian Physicians have chosen specialty training routes such as multi-year programs in traditional East Asian medicine.
• The **Canadian Academic Consortium on Integrative Healthcare Education (CACIHE)** began in 2002 to develop Complementary and Alternative Medicine in Undergraduate Medical Education. CACIHE is a national network of educators associated with Canada’s 17 medical schools collaborating to develop core competencies and content about complementary therapies for undergraduate medical education.^9^ It is currently on hiatus during the COVID pandemic.
• **Provincial Medical Associations.** Of fifteen medical associations in Canada, two have designated sections for Complementary and Integrative Medicine^10^: The Ontario Medical Association Medical Interest Group for Complementary and Integrative Medicine and the Doctors of Nova Scotia Section for Integrative and Complementary Medicine. These sections of the Medical Associations were formed to represent and advocate for IM physicians’ unique position within the medical profession.

## Discussion

**Figure 1.**
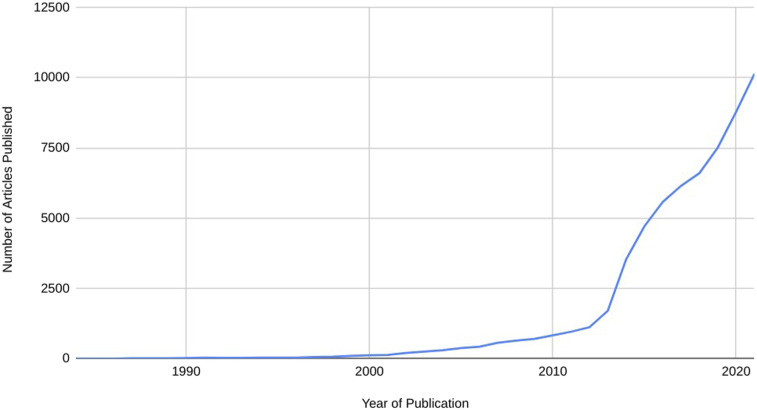
NIH PUB MED Search for “Integrative Medicine”.

The growth of CAM utilization by Canadian patients has occurred with limited engagement by Canadian physicians and Canadian medical institutions and to a lesser extent in Canada than it has in the United States. A number of factors have contributed to this:

(1) **Most of the training programs are in the United States.** This reduces accessibility to Canadian physicians. The virtual fellowships and training programs are also priced in U.S. dollars making them less affordable.
(2) **There are only rwo Canadian IM centres**^1^** affiliated with academic health centres, neither offering clinical services. and no IM centres in Canadian hospital systems.** This may be because neither the Royal College of Physicians and Surgeons of Canada or the College of Family Physicians of Canada recognize the ABOIM and therefore IM as a specialty. As a result, provincial health plans do not reimburse physicians for integrative medicine services. In contrast, there are IM centres and clinics in the United States offering services that are often reimbursed by insurance providers.
(3) **There is a perception by the conventional medicine community that Complementary and Integrative Medicine is not evidenced based.** In fact, here has been a rapid growth of published research in IM since 1990 (see Figure 1). 10% of the Cochrane reviews are related to CAM. The NCCIH conducts and funds research in Complementary and Integrative Health and along with the Canadian Institute of Health have invested more than $1.3B in research funding over the past decade.^11^ The quality of this research has been similar to conventional medicine research.^12^

## Conclusion

Canadians are increasingly using CAM therapies and the medical community needs to be able to better advise (or provide) these services to their patients. We suggest that official recognition of IM would better serve the Canadian public. This could be accomplished by two approaches. (1) Adding a “Certificate of Added Competence in Family Medicine” as a new domain of care, and (2) establishing an “Area of Focused Competence” designation through the Royal College of Physicians and Surgeons of Canada (RCPSC). These programs would enhance a physician’s practice, and would be available to family physicians and general practitioners. Priority topics and key features for the assessment of competence could be created in cooperation with academic experts in IM and working groups at the College of Family Physicians of Canada (CFPC) and the RCPSC.

Based on the experience in the United States and Europe, we believe that recognition through the CFPC and/or the RCPSC would support educational opportunities in Canada that would benefit physicians and their patients. Formal surveys of patient demand and practitioner training in Canada and elsewhere would provide feedback to guide the development of educational offerings.

## Note

1. University of Saskatchewan, Centre for Integrative Medicine and University of Toronto, Endowed Chair in Integrative Medicine.

## Notes

1 University of Saskatchewan, Centre for Integrative Medicine and University of Toronto, Endowed Chair in Integrative Medicine.
